# Two-year outcomes of PRESERFLO MicroShunt implantation in a real-world heterogeneous glaucoma cohort: a retrospective single-center study

**DOI:** 10.3389/fopht.2026.1835844

**Published:** 2026-09-01

**Authors:** Anina Zeier, Markus Tschopp, Daniel Barthelmes, Anthia Papazoglou

**Affiliations:** 1 Ophthalmology Department, Cantonal Hospital of Aarau, Aarau, Switzerland; 2 University of Zurich, Zurich, Switzerland; 3 University of Bern, Bern, Switzerland; 4 Ophthalmology Department, Inselspital Bern, Bern, Switzerland; 5 Ophthalmology Department, University Hospital of Zurich, Zurich, Switzerland

**Keywords:** glaucoma, intraocular pressure, MicroShunt, minimally invasive bleb surgery, mitomycin C, PreserFlo

## Abstract

**Purpose:**

To evaluate intraocular pressure (IOP), medication burden, best-corrected visual acuity (BCVA), and safety outcomes after *ab externo* PRESERFLO MicroShunt (PreserFlo) implantation in routine clinical practice.

**Methods:**

This retrospective single-center study included patients undergoing PreserFlo implantation (standalone or combined with phacoemulsification) between 2019 and 2022. IOP, BCVA, and glaucoma medications were collected preoperatively and postoperatively (day 1, week 1, month 1, year 1, and year 2). Hypotony was defined as IOP ≤ 5 mmHg. Complete success at 2 years required IOP ≤ 21 mmHg with ≥20% reduction from baseline without glaucoma medication.

**Results:**

Eighty-one eyes of 74 patients (mean age 73.2 years) were included. Mean baseline IOP was 24.0 ± 10.2 mmHg, decreasing to 12.5 ± 3.8 mmHg at year 1 (p < 0.0001) and 13.5 ± 3.4 mmHg at year 2 (p = 0.0005). Mean BCVA was 0.63 ± 0.30 at baseline, 0.70 ± 0.31 at year 1, and 0.73 ± 0.27 at year 2, indicating stable visual acuity during follow-up. Mean IOP reduction was 39.6% at year 1 and 33.8% at year 2; 75.6% and 73.3% achieved ≥20% IOP reduction at years 1 and 2, respectively. Medications decreased from 3.09 ± 1.02 to 0.44 ± 0.97 (year 1) and 0.45 ± 0.85 (year 2), and their medication-free rates were 80.5% and 73.3%, respectively. Complications occurred in 55.6% of eyes; 27.2% required postoperative interventions (needling 20.9%); 15.0% required further glaucoma surgery. Complete success rates were 60.2% (year 1) and 52.0% (year 2).

**Conclusions:**

PreserFlo implantation provided sustained mid-term IOP reduction with substantial medication sparing and stable BCVA.

## Introduction

Glaucoma is a leading cause of irreversible blindness, and its global burden is projected to rise substantially, exceeding 100 million affected individuals by 2040 (1). Intraocular pressure (IOP) is the only proven modifiable risk factor, and sustained IOP lowering remains the central therapeutic strategy to slow disease progression (2–4).

When IOP remains insufficiently controlled despite topical therapy and selective laser trabeculoplasty, surgery becomes necessary. Trabeculectomy and tube shunts provide robust pressure lowering, but they require intensive postoperative care and may be associated with significant complications (5). Minimally invasive glaucoma surgery (MIGS) aims to reduce surgical morbidity; within this spectrum, minimally invasive bleb-forming surgery (MIBS) can achieve larger and more durable IOP reductions and may therefore be suitable for patients with moderate-to-advanced disease (6–8).

The PRESERFLO MicroShunt (PreserFlo) is an *ab externo* subconjunctival drainage device manufactured from poly(styrene-*block*-isobutylene-*block*-styrene) (9, 10). Its lumen dimensions are designed to provide controlled aqueous outflow based on the principles of fluid dynamics, aiming to reduce the risk of chronic hypotony while enabling bleb formation, particularly when combined with antimetabolite therapy (9–11). Published cohorts and comparative studies report clinically meaningful IOP reduction and medication sparing, alongside an evolving understanding of the postoperative bleb management required to maintain long-term function (12–18).

Here, we report 2-year outcomes of PreserFlo implantation in a heterogeneous real-world cohort, focusing on IOP control, medication burden, visual acuity, complications, and reinterventions.

## Methods

This retrospective single-center study included consecutive patients undergoing PreserFlo implantation between 2019 and 2022 at the Cantonal Hospital Aarau (KSA), Switzerland. The study adhered to the tenets of the Declaration of Helsinki and complied with applicable national legal and regulatory requirements; ethics approval was granted (approval ID: 2023-00488). Only eyes of patients with documented signed general consent were included. Surgeries were performed by five experienced glaucoma surgeons as standalone procedures or combined with phacoemulsification cataract extraction.

### Data collection and definitions

Baseline IOP (mmHg), best-corrected visual acuity (BCVA; Snellen), and the number of glaucoma medications were recorded at the visit when surgery was indicated. Follow-up assessments were obtained on postoperative day 1, week 1, month 1, year 1, and year 2. Primary analyses at year 1 and year 2 included only eyes with data available at the respective time point. Last available follow-up was not used to replace missing year 1 or year 2 data. Fixed-combination drops were counted as two medications.

Hypotony was defined as IOP ≤ 5 mmHg. Hyphema was defined as any visible blood in the anterior chamber (no grading). Documented complications included IOP rise, hypotony, choroidal detachment, hyphema, and bleb encapsulation/fibrosis. Interventions included slit-lamp needling (with MMC), anterior chamber viscoelastic injection, anterior chamber washout, bleb revision, cyclophotocoagulation, and additional glaucoma surgery.

### Outcomes

Complete success at 2-year follow-up was defined as IOP ≤ 21 mmHg with ≥20% reduction from baseline without glaucoma medications, while qualified success remains defined as the same IOP criterion with glaucoma medication (6, 13, 14, 17, 19). Needling and anterior chamber viscoelastic injection were not classified as failures, as these were considered postoperative management procedures for expected bleb-related or hypotony-related events after filtering surgery rather than glaucoma surgical failure. Failure was defined as the need for additional glaucoma surgery (e.g., bleb revision, second microshunt implantation, or cyclophotocoagulation) or loss of light perception.

### Surgical technique and postoperative care

A standard *ab externo* approach with conjunctival/Tenon dissection was used. Intraoperative mitomycin C (MMC; 0.3–0.4 mg/mL) was applied subconjunctivally for 2 min using three soaked sponges to reduce fibroblast proliferation and postoperative scarring (20). The variation in MMC concentration reflected a change in institutional pharmacy preparation during the study period rather than individualized dose selection. The intracameral position of the microshunt was routinely verified intraoperatively using gonioscopy to confirm appropriate entry through the trabecular meshwork and to avoid corneal or iris contact (11, 21). If positioning was deemed suboptimal, the implant was explanted and re-implanted through a newly created scleral tract with a fresh entry point 3 mm posterior to the limbus, followed by repeat gonioscopic confirmation. This was performed to optimize long-term implant positioning and reduce the risk of later anterior chamber structure contact with possible endothelial cell loss. Postoperative therapy included short-term topical antibiotics and long-term (tapered for at least 3 months) topical steroids.

### Statistical analysis

Data were analyzed using GraphPad Prism (version 10.4.0). Continuous variables are presented as mean ± standard deviation (SD), and categorical variables as counts (percentages). Longitudinal changes in IOP, BCVA, and the number of glaucoma medications were analyzed using mixed-effects models fitted by restricted maximum likelihood, with time point as a fixed effect and repeated measurements matched within eyes. Sphericity was not assumed, and Geisser–Greenhouse correction was applied. Pairwise comparisons between baseline and postoperative time points were adjusted for multiple testing using Dunnett’s multiple comparisons test. p-Values <0.05 were considered statistically significant.

To address potential inter-eye correlation due to the inclusion of both eyes in seven patients, a sensitivity analysis was performed including only one eye per patient. In bilaterally included patients, the first operated eye was retained, resulting in a sensitivity cohort of 74 eyes from 74 patients. The mixed-effects analyses were repeated using the same model specifications as in the full cohort.

Complete and qualified surgical success were assessed using the Kaplan–Meier survival analysis. Complete success required IOP ≤ 21 mmHg with ≥20% reduction from baseline without glaucoma medication; qualified success used the same IOP criterion while allowing medication. Failure was assessed from postoperative month 1 onward and was defined as failure to meet the IOP criterion, additional glaucoma surgery, or loss of light perception. Reintroduction of glaucoma medication was additionally considered failure for complete success.

## Results

### Cohort characteristics

Seventy-four patients (81 eyes) underwent surgery; 36 (48.65%) were female, and the mean age was 73.2 years (range 40–86). Diagnoses included primary open-angle glaucoma (n = 47), pseudoexfoliation glaucoma (n = 21), secondary glaucoma (n = 11), narrow-angle glaucoma (n = 1), and congenital glaucoma (n = 1). Fifty-five surgeries were standalone, and 26 were combined with phacoemulsification. These demographic characteristics are displayed in **Table 1**. Implant placement was superotemporal in 76 cases and superonasal in 5; seven patients underwent bilateral surgery. The number of eyes analyzed at each time point was as follows: baseline, n = 81; day 1, n = 81; week 1, n = 78; month 1, n = 65; year 1, n = 41; and year 2, n = 30. The mean follow-up duration was 353.9 days, corresponding to approximately 11.6 months. The median follow-up was 356.5 days.

**Table 1:** Baseline demographic, ocular, and surgical characteristics of eyes undergoing PRESERFLO MicroShunt implantation.

Baseline demographic characteristics	Value
N (eyes)	81
Age at surgery [mean (SD)]	73.2 (9.12)
Female sex (%)	36 (48.65)
Operated eye (%), right	44 (54.3)
Phakic lens status (%)	33 (40.7)
Glaucoma type (%)	
- POAG	47 (58.0)
- PXG	21 (25.9)
- Narrow angle	1 (1.2)
- Secondary	11 (13.58)
- Congenital	1 (1.2)
Clinical proxies of glaucoma severity/disease burden	
- Baseline IOP, mean ± SD, mmHg	24.0 ± 10.2
- Number of glaucoma medications, mean ± SD	3.09 ± 1.02
- Previous ocular surgery (%)	17 (20.98)
- Baseline BCVA, mean ± SD, decimal Snellen	0.626 ± 0.302
- Previous laser therapy (%)	17 (20.98)
- Prior trabeculectomy (%)	8 (9.87)
- Prior XEN (%)	5 (6.17)
- Prior canaloplasty (%)	1 (1.23)
- Prior cyclophotocoagulation (%)	2 (2.46)
Surgery type (%)	
- PreserFlo	55 (67.9)
- PreserFlo + Phaco	26 (32.0)

Standardized visual field indices and OCT-based structural parameters were not available in a sufficiently complete and uniform manner for all eyes and were therefore not included in the main analysis. Data are presented as mean (SD), median (range), or number of eyes (%), as appropriate. BCVA was recorded as decimal Snellen acuity.

BCVA, best-corrected visual acuity; IOP, intraocular pressure; POAG, primary open-angle glaucoma; PXG, pseudoexfoliation glaucoma.

### Intraocular pressure

**Figure 1:**
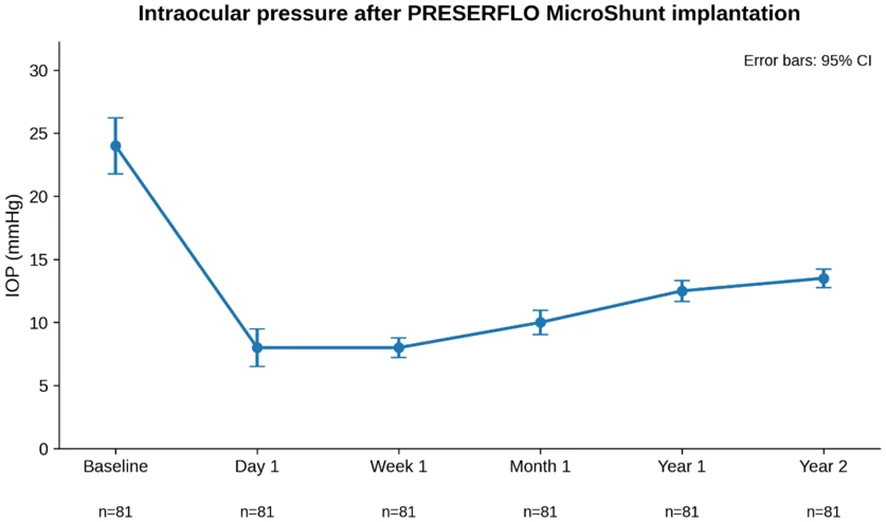
Mean intraocular pressure before and after PRESERFLO MicroShunt implantation. Mean IOP (mmHg) is shown at baseline and at postoperative day 1, week 1, month 1, year 1, and year 2. Error bars indicate 95% CI. IOP changed significantly over time. Compared with that at baseline, IOP was significantly lower at day 1, week 1, month 1, and year 1, all adjusted p < 0.0001, and at year 2, adjusted p = 0.0005. IOP, intraocular pressure.

Mean baseline IOP was 24.0 ± 10.2 mmHg (range 10–62). Mean IOP decreased to 8.0 ± 6.8 mmHg (day 1), 8.0 ± 3.6 (week 1), 10.0 ± 4.4 (month 1), 12.5 ± 3.8 (year 1), and 13.5 ± 3.4 (year 2) (**Figure 1**). In the mixed-effects model, IOP changed significantly over time, F(2.269, 131.1) = 72.26, p < 0.0001. Compared with that at baseline, IOP was significantly lower at all postoperative time points after performing Dunnett’s adjustment: day 1, week 1, month 1, and year 1 all adjusted p < 0.0001, and year 2 adjusted p = 0.0005.

Mean IOP reduction was 39.6% at year 1 and 33.8% at year 2; 75.6% and 73.3% achieved ≥20% IOP reduction at years 1 and 2, respectively.

Standalone PRESERFLO implantation was performed in 55 eyes and combined PRESERFLO implantation with phacoemulsification in 26 eyes. In the standalone group, mean IOP was 11.8 mmHg at year 1 and 13.2 mmHg at year 2. In the combined surgery group, mean IOP was 13.9 mmHg at year 1 and 14.3 mmHg at year 2. The difference between groups was not statistically significant at either year 1 or year 2 (p = 0.0869 and p = 0.3378, respectively).

### Glaucoma medications

**Figure 2:**
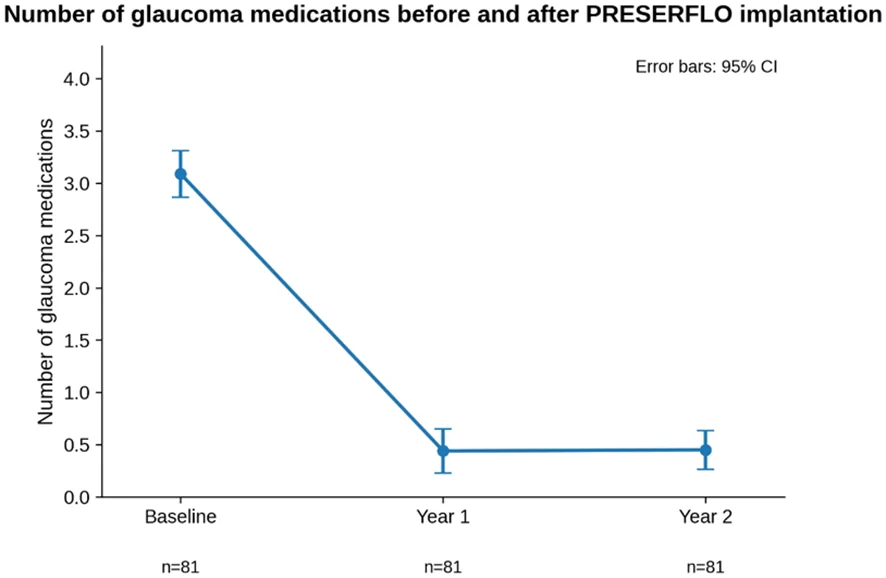
Mean number of glaucoma medications before and after PRESERFLO MicroShunt implantation. Mean number of glaucoma medications is shown at baseline and at postoperative year 1 and year 2. Error bars indicate 95% CI. Medication-free rates were 80.5% at year 1 and 73.3% at year 2. Compared with that at baseline, the number of glaucoma medications was significantly lower at year 1 and year 2, both adjusted p < 0.0001.

Mean medications decreased from 3.09 ± 1.02 (range 0–4) preoperatively to 0.44 ± 0.97 at year 1 and 0.45 ± 0.85 at year 2 (**Figure 2**). Medication burden changed significantly over time in the mixed-effects model, F(1.610, 56.36) = 140.7, p < 0.0001. Compared with that at baseline, the number of glaucoma medications was significantly reduced at year 1 and year 2 after performing Dunnett’s adjustment, both adjusted p < 0.0001. Medication-free IOP control was achieved in 80.5% at year 1 and 73.3% at year 2. Reduced long-term drop burden may mitigate preservative-associated conjunctival changes relevant to bleb surgery outcomes (22).

### Visual acuity

Mean preoperative BCVA was 0.63 ± 0.30 (range 0.05–1.25). BCVA decreased transiently on day 1 (0.38 ± 0.26) and improved thereafter (week 1, 0.46 ± 0.28; month 1, 0.52 ± 0.26). At year 1 and year 2, mean BCVA was 0.70 ± 0.31 and 0.73 ± 0.27, respectively. In the mixed-effects model, BCVA changed significantly over time, F(3.277, 161.9) = 26.11, p < 0.0001. *Post-hoc* Dunnett’s comparisons showed a significant decrease in BCVA at day 1 and week 1 compared with baseline, both adjusted p < 0.0001. BCVA at month 1, year 1, and year 2 did not differ significantly from baseline after adjustment (month 1 adjusted p = 0.3395, year 1 adjusted p = 0.0746, and year 2 adjusted p = 0.0920). No severe visual loss attributable to microshunt implantation was observed.

### Sensitivity analysis accounting for bilateral cases

In the one-eye-per-patient sensitivity analysis, the longitudinal findings remained consistent with the full-cohort analysis. IOP remained significantly changed over time, F(2.236, 118.1) = 69.73, p < 0.0001, with significantly lower IOP at all postoperative time points compared with baseline after performing Dunnett’s adjustment. BCVA also remained significantly changed over time, F(3.415, 153.7) = 23.98, p < 0.0001; *post-hoc* comparisons confirmed a significant early decrease at day 1 and week 1, whereas BCVA at month 1, year 1, and year 2 did not differ significantly from baseline. Thus, the exclusion of second eyes from bilaterally included patients did not materially affect the main conclusions.

### Complications and reinterventions

Complications occurred in 45/81 eyes (55.6%). The most frequent were hypotony (40%), IOP rise (22%), hyphema (15%), choroidal detachment (12%), and bleb encapsulation/fibrosis (10%). Hypotony, hyphema, and choroidal detachment predominated early, whereas bleb encapsulation/fibrosis emerged later (**Table 2**). Twenty-two eyes (27.2%) required postoperative interventions (**Table 3**): needling in 20.9% (18 needlings), anterior chamber viscoelastic injection (7 instances), and anterior chamber washout (1 case). Further glaucoma surgery was required in 12 eyes (15.0%), including cyclophotocoagulation (2.46%), bleb revision (9.87%), and second PreserFlo implantation (3.72%).

**Table 2:** Postoperative complications after PRESERFLO MicroShunt implantation, including temporal pattern of occurrence.

Follow-up	IOP rise	Hypotony ≤ 5 mmHg	Choroidal detachment	Hyphema	Encapsulation and/or fibrosis
1–3 days	3	23	0	12	0
1 week	1	15	8	4	0
1 month	8	5	5	0	0
1 year	8	0	0	0	9
2 years	4	0	0	0	2
Total	24	43	13	16	11
**(%)**	**22%**	**40%**	**12%**	**15%**	**10%**

Values are presented as number of eyes (%). Early complications included hypotony, hyphema, and choroidal detachment, whereas bleb encapsulation/fibrosis was typically observed later during follow-up. Multiple complications could occur in the same eye.

IOP, intraocular pressure.

**Table 3:** Postoperative interventions and secondary glaucoma procedures after PRESERFLO MicroShunt implantation.

Type of retreatment/intervention	Frequency	Percentage of retreatments/interventions (%)	Percentage of operated eyes (%)	Number of eyes
Cyclophotocoagulation	3	7	2.46%	2
Needling	18	45	22.22%	18
Bleb revision	8	19	9.87%	8
2nd PreserFlo implantation	4	10	3.72%	3
Viscoelastic	7	17	6.16%	5
Anterior chamber washout	1	2	1.23%	1
**Total**	**41**	**100%**	**45.7%**	**37**

Values are presented as number of eyes (%) or number of interventions, as appropriate. Postoperative interventions included slit-lamp needling with mitomycin C, anterior chamber viscoelastic injection, and anterior chamber washout. Secondary glaucoma procedures included bleb revision, cyclophotocoagulation, and second PRESERFLO implantation. More than one intervention could be performed in the same eye.

### Kaplan–Meier analysis

**Figure 3:**
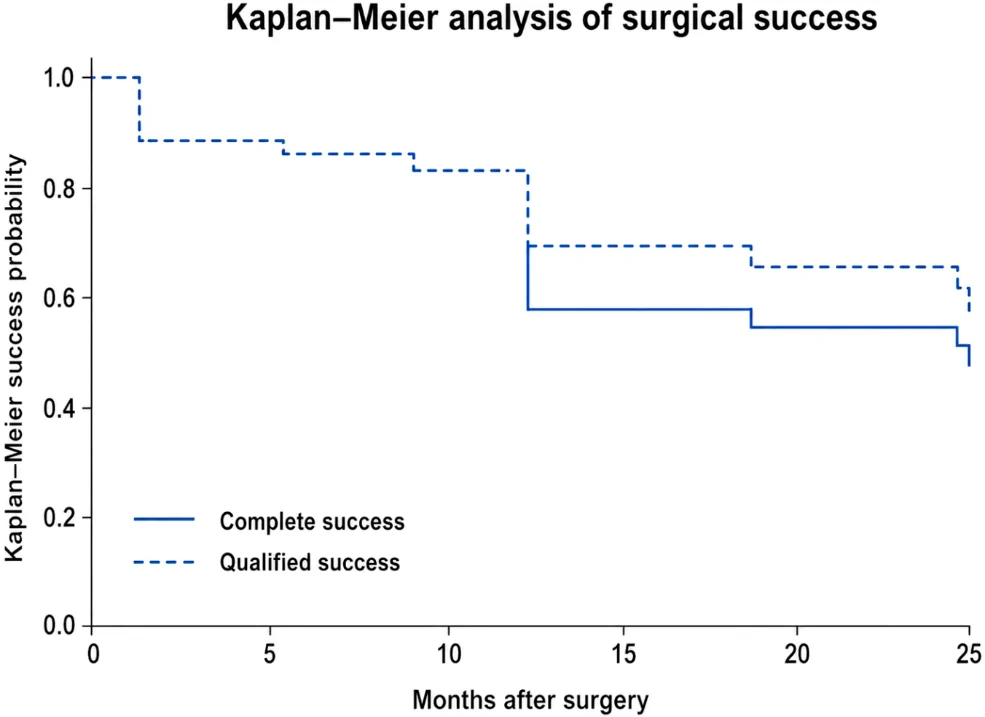
Kaplan–Meier analysis of complete and qualified surgical success after PRESERFLO MicroShunt implantation. Kaplan–Meier curves show the probability of complete and qualified surgical success over 24 months (81 eyes). Complete success was defined as IOP ≤ 21 mmHg with ≥20% reduction from baseline without glaucoma medication, and qualified success using the same IOP criterion while allowing medication. Failure criteria were evaluated from postoperative month 1 onward. Additional glaucoma surgery was considered a failure in both analyses; reintroduction of glaucoma medication was considered a failure only for complete success. IOP, intraocular pressure.

The Kaplan–Meier analysis was performed for complete and qualified surgical success in the full cohort (81 eyes) (see **Figure 3**). The estimated complete success probability was 60.2% at 12 months and 52.0% at 24 months, while the estimated qualified success probability was 70.5% and 61.3%, respectively. The estimated qualified success probability was 70.5% at 12 months and 61.3% at 24 months. In a one-eye-per-patient sensitivity analysis, the Kaplan–Meier estimates remained comparable, indicating that the inclusion of both eyes in seven patients did not materially affect the surgical success estimates.

## Discussion

In this real-world retrospective cohort, *ab externo* PreserFlo implantation provided substantial and durable IOP lowering over 2 years. Mean IOP decreased from 24.0 mmHg to 12.5 mmHg at 1 year (−39.6%) and 13.5 mmHg at up to 2 years (−33.8%), which is in line with previous PreserFlo reports showing reductions from 23.8 to 10.7 mmHg at 1 year and 11.9 mmHg at 2 years, from 23.4 to 14.7 mmHg at 1 year, and sustained low-teen IOP values up to 5 years (12–17). The modest IOP increase between year 1 and year 2 is compatible with progressive wound healing and subconjunctival fibrosis, which remains a key determinant of long-term bleb performance (20, 23). Exploratory subgroup analysis did not show a statistically significant difference in postoperative IOP between standalone PRESERFLO implantation and PRESERFLO combined with phacoemulsification; however, this finding should be interpreted cautiously given the non-randomized retrospective design and limited subgroup size.

Medication burden decreased markedly from 3.0 to ~0.45 at both 1 and 2 years, with medication-free rates of 80.5% and 73.3%, respectively, mirroring prior PreserFlo cohorts in which mean medication use fell from 2.4 to 0.3 at 1 year and 0.4 at 2 years, and from 2.1 to 0.5 at 2 years, with 73.8% of patients being medication-free; notably, our medication-free rates were at the upper end of previously reported ranges (13–17). This is clinically important because complex regimens are associated with poorer adherence, ocular surface morbidity, reduced quality of life, and higher long-term cost (24, 25). BCVA remained stable at 1 and 2 years, consistent with published functional safety data (13, 15, 16).

Postoperative complications were frequent, but overall consistent with the known safety profile of bleb-forming surgery. Hypotony was observed in 40% of eyes, which is higher than in several published PRESERFLO series (21, 26, 27). However, it was predominantly short-lived early numerical hypotony rather than persistent or vision-threatening hypotony. The underlying explanation for this finding remains uncertain. A possible contributor may have been the intraoperative need, in selected cases, to explant and reimplant suboptimally positioned devices through a new scleral tract. While additional scleral entry sites could theoretically facilitate transient early overfiltration, this remains speculative and should be interpreted cautiously. Hyphema was observed early and appeared more frequent than in some cohorts, likely reflecting differences in definitions/grading and case mix (12, 14, 17, 27). The postoperative intervention burden in our cohort was clinically relevant: 27.2% of eyes required additional procedures, including needling in 20.9%, and 15.0% underwent further glaucoma surgery. These rates were at the upper end of previously reported ranges in the PreserFlo series. For example, Armstrong et al. reported needling in 15.1%, revisions in 7%, and reoperations in 3% over 3 years, whereas Batlle et al. described a low rate of postoperative interventions in their long-term extension cohort. Such differences likely reflect not only case mix and follow-up duration but also variation in definitions and center-specific postoperative algorithms. In our analysis, slit-lamp needling and anterior chamber viscoelastic injection were considered part of postoperative management and were not classified as failures, whereas secondary glaucoma procedures such as bleb revision, cyclophotocoagulation, or second PRESERFLO implantation were classified as surgical failure. Taken together, these findings reinforce that PreserFlo is not a “low-maintenance” procedure and that outcomes depend on structured bleb surveillance and timely management of subconjunctival scarring (13, 14, 17, 21). Complete success was 60.2% after 1 year and 52.0% after 2 years, while their qualified success was 70.5% and 62.3%, respectively (13, 14, 17, 19).

Limitations include the retrospective design, the heterogeneous real-world cohort composition, variable thresholds for reintervention, potential attrition bias, lack of Optical Coherence Tomography (OCT)/visual field endpoints, and absence of a comparator arm. In particular, follow-up completeness decreased over time, with 2-year data available for 30 of 81 eyes. Therefore, the 2-year estimates should be interpreted cautiously, as outcomes among eyes with longer follow-up may not fully represent the entire operated cohort. Another limitation is that MMC concentration varied between 0.3 and 0.4 mg/mL due to a change in institutional pharmacy preparation over time. As the exact timing of this change could not be reconstructed retrospectively with sufficient certainty, individual eyes could not be reliably assigned to one MMC concentration group, precluding a valid comparison of outcomes or complication rates according to MMC concentration. Finally, the cohort included different glaucoma subtypes, standalone and combined phacoemulsification procedures, and surgeries performed by five surgeons, but the sample size was insufficient for robust subgroup analyses. Nonetheless, these results provide pragmatic real-world estimates: PreserFlo can deliver meaningful, sustained IOP reduction with substantial medication sparing, but requires an infrastructure for postoperative bleb optimization. Prospective comparative studies with standardized algorithms and stratification by MMC dose, glaucoma subtype, baseline IOP, and prior surgery are warranted to refine patient selection and optimize long-term outcomes (8, 28).

## Conclusions

PreserFlo implantation provided sustained 2-year IOP reduction with substantial medication sparing and stable BCVA in routine clinical practice. The safety profile was consistent with that expected for bleb-forming surgery, with postoperative complications and reinterventions that were frequent but generally manageable. Overall, PreserFlo appears to be an effective surgical option in appropriately selected patients, provided that postoperative surveillance, bleb optimization, and timely reintervention are integrated into clinical care pathways.

## Data Availability

The raw data supporting the conclusions of this article will be made available by the authors, without undue reservation.
